# Case report: GLP1RA for the treatment of diabetes in liver transplanted people. Do they increase the risk of pancreatitis?

**DOI:** 10.3389/fendo.2024.1392371

**Published:** 2024-05-08

**Authors:** Valeria Grancini, Irene Cogliati, Alessia Gaglio, Veronica Resi, Emanuela Orsi

**Affiliations:** Endocrinology Unit, Fondazione IRCCS Ca’ Granda – Ospedale Maggiore Policlinico Milano, Milan, Italy

**Keywords:** GLP1RAs, pancreatitis, liver transplantation, incretin agents, acute pancreatitis

## Abstract

The incidence of acute pancreatitis (AP) in liver transplanted people is reported to be 1.5-8%. On the other hand, the evidence for a causal relationship between glucagon-like peptide 1 receptor agonists (GLP1RAs) and pancreatitis in people with type 2 diabetes is still weak. In addition, there are currently no data on a possible increased risk of AP in liver-transplanted individuals with diabetes treated with GLP1RAs. In a population of liver-transplanted individuals with diabetes receiving GLP1RA-based therapy, we reported an incidence of AP of 3.0% (two subjects). No cases were reported in liver-transplanted individuals with diabetes receiving SGLT2 inhibitors, insulin or metformin, neither in kidney or lung-transplanted patients treated with GLP1RAs. In both patients with AP, the only additional risk factor for its development was a history of re-transplantation (liver or combined kidney/liver). For this reason, we suggest particular caution when considering GLP1RAs-based therapies in liver transplanted patients with multiple risk factors for AP, such as a history of repeated and complex abdominal surgery.

## Introduction

Acute pancreatitis (AP) is one of the most common gastrointestinal diseases (1, 2). Its incidence varies from 5-73 cases/10000 individuals (2, 3). Overall mortality is up to 5% (4) and gallstones, alcohol abuse, hypertriglyceridemia, endoscopic retrograde cholangiopancreatography (ERCP), genetics, drugs and pancreatic duct injuries (5–9) are well established risk factors. The epidemiology of AP is well characterized in the general population, but more conflicting data are available for solid organ transplanted individuals. Graham D. et al, in a recent review, reported an incidence of 1-35%, with the highest rates in kidney and intestinal transplantation in pediatric populations (10).

In organ transplanted patients, a lower age (11), hypercalcemia (12), alcohol consumption (13), gallstones (12), congenital abnormalities of the pancreas (12), viral infections (CMV; VZV; HBV) (12) and pre-transplant history of AP (11) have been reported as major risk factors. Moreover, several immunosuppressant therapies such as tacrolimus, cyclosporine, azathioprine, mycophenolate mofetil have been mentioned as possible risk factors (11, 14–16).

Actually, the incidence of AP in liver transplanted people is reported to be 1.5-8% (17–19) and a recent study by Hujova A. and colleagues reported, as well demonstrated risk factors, previous intra-abdominal surgery, HBV infection and post-transplant biliary complications needing ERCP (20).

GLP1 RAs have become first line treatments for DM2 in recent years, due to their widely recognized beneficial effects on glycemic control, their cardio-renal protection and weight loss (21–26). For all these reasons mentioned above, the 2024 American Diabetes Association Guidelines suggest that, in adults with type 2 diabetes and established/high risk or atherosclerotic cardiovascular disease (ASCVD), the treatment plan should include a GLP1RA agent with proven cardiovascular protection (27).

In 2008, the US Food and Drug Administration (FDA) suggested a potential association between exenatide and AP referring to 30 case reports of AP (28). Since then, warnings about the risk of AP have continued to grow, resulting in a global discussion on this issue (29, 30). Several documents have been published discussing the possible risk of AP in people receiving incretin-based therapies (31–35).

A potential link between GLP1RAs and AP was hypnotized basing on data from experimental studies on animals. The possible underlying mechanism proposed was that the chronic hyperstimulation of exocrine pancreas by GLP1RAs. Several pharmacovigilance reports have endorsed this hypothesis, while retrospective observational studies didn’t provide univocal answers on this topic, with the main part of studies disproving this relationship (36).

Actually, a causal association between incretin-based drugs and AP has not been definitively confirmed and a recent meta-analysis of 43 randomized controlled trials found no clear evidence of a strict relationship between AP in people with type 2 diabetes and GLP1RAs (37).

Despite the proven higher risk of AP in transplanted individuals and given the previous alerts on the relationship between GLP1RAs use and AP (although if never confirmed), no clear indications on the use of these drugs in liver-transplanted people with diabetes. Finally, no data are currently available on the potential increased risk of AP in liver-transplanted individuals with diabetes and treated with GLP1RAs.

On the one hand, such evidences could represent a relative contraindication to their use. On the other hand, abstention from it would deny the proven cardiovascular protection, offered by these drugs, in a high-risk population.

Data displayed in this case report are from our registry on liver transplanted patients, currently followed at our Clinic. The research protocol was approved by the 129 Ethics Committee of the IRCCS Ca’ Granda – Ospedale Maggiore Policlinico Foundation (Prot. n. 516) and has been registered on ClinicalTrials.gov (Identifier nr: NCT02038571).

## Case report

251 liver transplanted individuals with diabetes were followed from 2016 to 2024 at the Endocrinology Unit of Fondazione IRCCS Ca’ Granda – Ospedale Maggiore Policlinico of Milan, Italy; among them, 66 patients started a GLP1RA-based therapy.

The choice of the therapeutic scheme was made according to current Standards of Care (27), and the most frequent reasons to start or switch to a GLP1RA-based therapy were the high cardio-vascular (CV) risk, a previous CV event or the need to lose weight in overweight/obese individuals. During this 8-years follow-up period, 2 patients from the GLP1RAs-treated group developed AP (3%).

A third patient, male, 66 years old, had to reduce the dose of dulaglutide from 1.5 to 0.75 mg/week because of a > 3-fold increase in pancreatic enzymes (amylase levels up to 166 UI/L lipase levels up to 249 UI/L).

### Case 1: male, 66 years old

Patient nr.1 underwent liver transplantation for cirrhosis caused by secondary sclerosing cholangitis after echinococcosis in January 2016 and re-transplantation with Roux-en-Y hepaticojejunostomy technique for liver insufficiency after hepatic artery thrombosis (HAT) in April 2016. He developed post-transplant diabetes mellitus in the immediate post-transplant period and started insulin therapy by multiple daily injection scheme. In 2019, due to the presence of microalbuminuria, he was switched to basal insulin (degludec) 14 IU/day and canagliflozin 300 mg/day, as per guidelines, and in October 2022, dulaglutide 1.5 mg/week was added. He was on immunosuppressant therapy with tacrolimus and mycophenolate mofetil. Outpatient visits were performed every 6 months. At the visits, we monitored glucose profiles, HbA1c levels, renal function, lipid profile and amylase and lipase levels, which always remained within normal ranges. 10 months after starting GLP1RA-based therapy, the patient presented to the emergency department of our hospital with abdominal pain, fever and vomiting. A nasopharyngeal swab revealed a concomitant infection with Covid-19. During hospitalization, amylase and lipase levels increased from normal ranges to 219 UI/L and 298 UI/L, respectively. Computed tomography (CT) revealed an Intraductal Papillary Mucinous Neoplasm (IPMN) and magnetic resonance (MR) showed a typical pattern of AP.

Dulaglutide and Canagliflozin were discontinued and the patient was discharged on insulin therapy with an MDI regimen. At the last outpatient visit to our clinic amylase and lipase levels returned to normal ranges and canagliflozin was reintroduced.

### Case 2: male, 61 years old

Patient nr.2 was affected by autosomal dominant polycystic kidney disease (ADPKD). He underwent left nephrectomy in 2002, kidney transplantation in 2007, right nephrectomy in 2009 and combined kidney-liver transplantation in January 2019. Liver transplantation was performed according to Starzl technique, with end-to-end anastomosis between the bile ducts.

He developed diabetes in 2019 and started insulin therapy by multiple daily injection scheme. He gradually reduced the total daily dose according to the steroid tapering. In 2022 he was switched to dulaglutide 1.5 mg weekly, due to the presence of macroalbuminuria and inadequate glomerular filtration rate for SGLT2 drugs (c-GFR, calculated according to the CKD-EPI formula: 19 mg/dl/m^2^) (38).

He was on immunosuppressant therapy with prednisone, tacrolimus and mycophenolate mofetil. Outpatient visits were performed every 6 months. As in Case 1, glucose profiles, HbA1c levels, lipid profile and amylase and lipase levels were continuously monitored and remained within normal ranges. After 22 months of GLP1RA-based therapy, the patient developed AP, with amylase and lipase levels rising from normal ranges to 433 UI/L and 1057 UI/L, respectively.

Dulaglutide was discontinued and the patient was discharged without any pharmacological treatment for diabetes. At his last outpatient visit to our clinic amylase and lipase levels returned to normal ranges and HBA1c was 45 mmol/mol so we didn’t start any additional therapy, despite the known proteinuria.

**Figure 1:**
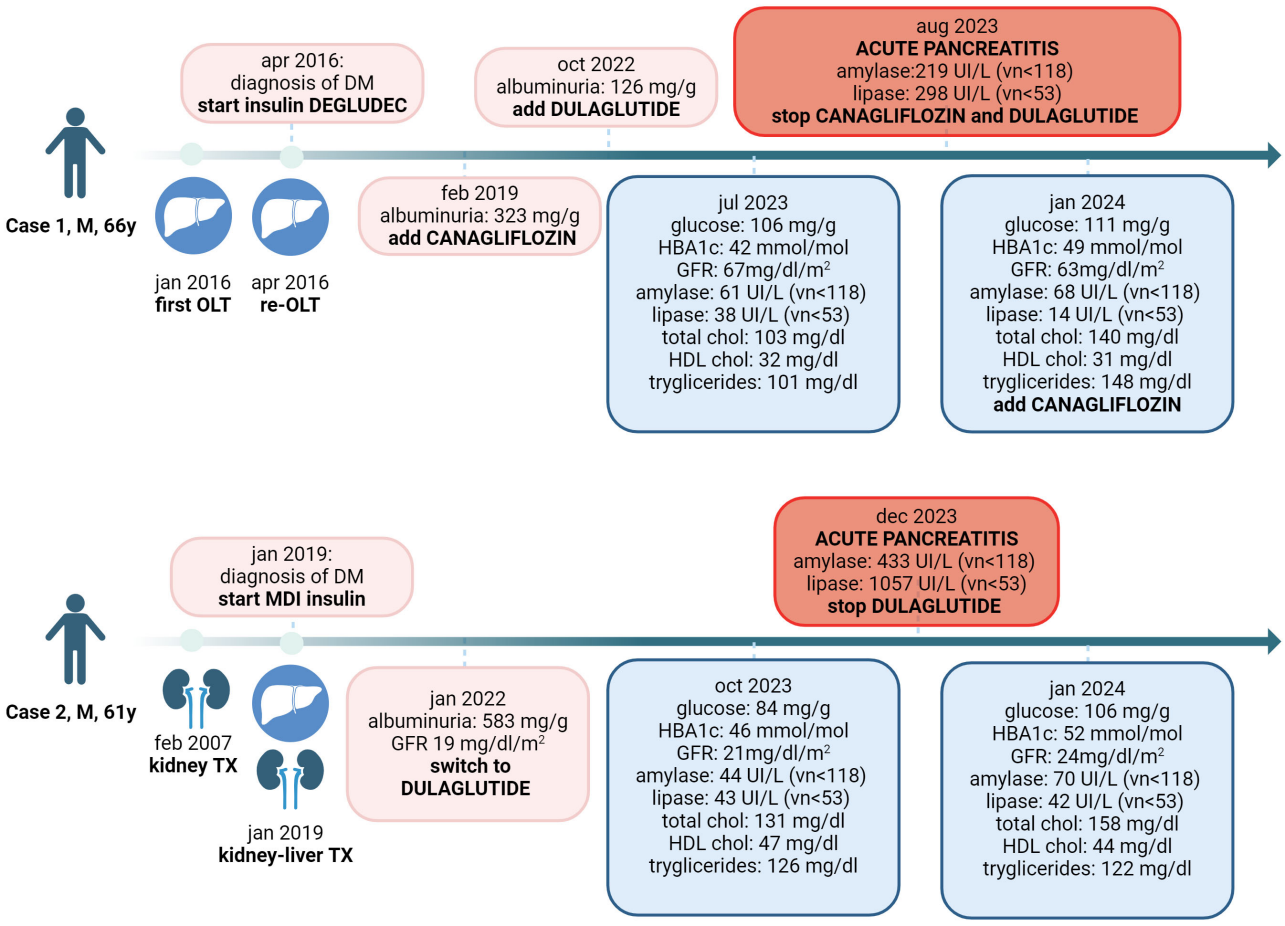
Timeline with the relevant clinical data for Case 1 and Case 2. M, male; OLT, orthotopic liver transplantation; DM, diabets mellitus; HbA1c, glycated hemoglobin; GFR, glomerular filtration rate; chol, cholesterol; HDL, high density lipoprotein; TX, transplantation.

The timeline with the relevant data for each patient is shown in **Figure 1**.

## Discussion

In our population of 66 liver transplant recipients with diabetes, we observed an incidence of AP of 3.0% in people taking a GLP1RA-based therapy. Conversely, we had no cases of AP among liver transplanted individuals who were managing their diabetes with SGLT2 inhibitors, metformin, insulin-based therapies or a combination of these molecules (42, 47, 51 and 38 individuals, respectively). Furthermore, 112 kidney-transplanted people with diabetes and 56 lung-transplanted people with diabetes regularly attend outpatients visits at our Clinic. We observed no cases of AP in kidney- and lung-transplanted individuals with diabetes who were on GLP1RA-based therapy (35 and 10 people, respectively).

We therefore investigated whether the coexistence of GLP1RAs-treated diabetes and liver transplantation could act synergistically as a risk factor for the development of AP.

As mentioned above, in both individuals with liver transplantation who developed AP, several conditions could be considered as potential risk factors for developing AP:

### Surgical factors

Repeated surgical interventions on the abdominal cavity and potential injuries/ischemic damage to the pancreatic and peripancreatic tissues are known risk factors for the development of AP. In addition, the use of Roux-en-Y loop choledochojejunostomy has been associated with an increased risk of AP (18).

In our population, Case 1 underwent liver re-transplantation after first graft failure and underwent a Roux-en-Y loop choledochojejunostomy during the second transplantation.

Case 2 underwent a combined kidney-liver transplantation after a hesitant first kidney transplant with graft failure.

### Infective causes

Infectious causes of AP, although rare, has been described in the general population; viral agents such as herpes virus and cytomegalovirus; bacterial agents as mycoplasma, legionella, leptospira, mycosis and parasitosis have been reported (39).

At the time of admission to our Hospital for AP, Case 1 presented with a positive nasopharyngeal swab for Covid-19: moreover, before transplantation, he suffered from secondary sclerosing cholangitis after echinococcosis and, during hospitalization for AP, he underwent lung biopsy with histopathological evidence of pulmonary echinococcosis.

### Concomitant medications

Several immunosuppressant therapies have been reported to be potential causes of pancreatitis (16) but, actually, data about their role as a risk factor for drug-induced AP after liver transplantation are still conflicting (18, 40). In our population, however, the immunosuppressant regimens used for both clinical cases didn’t differ from other liver-transplanted patients.

### Other risk factors

Alcoholic cirrhosis is one of the leading causes of liver transplantation in USA and Europe (41). Again, alcohol is the second most common risk factor for AP in the general population and the cause of 25% of post-liver transplant AP (42). In our case series, none of them had alcoholic cirrhosis and both reported total abstinence from alcohol.

Finally, hypertriglyceridemia is a further well demonstrated common cause of AP, and typically occurs in patients with concomitant uncontrolled diabetes, alterations in lipoprotein metabolism, alcohol abuse or concomitant medications (43). Despite concomitant immunosuppressant therapy with tacrolimus and the presence of diabetes and metabolic syndrome, both patients had normal lipid profiles and normal triglycerides levels (both patients were on statins and omega 3- based lipid-lowering therapy).

In **Table 1** are schematically reported the potential additional risk factor for AP for each patient.

**Table 1:** Potential additional risk factor for AP for each patient.

	Case 1	Case 2
**Surgical Factors:**	Liver re-transplantation Roux-en-Y hepaticojejunostomy	Combined kidney/liver re-transplantation
**Infective factors:**	Echinococcosis Covid-19 concomitant infection	none
**Drugs:**	Tacrolimus	Tacrolimus
**Other:**	None	none

In conclusion, in our liver-transplanted patients with diabetes treated with GLP1RAs, the incidence (3%) of AP didn’t differ from data reported in the current literature on the incidence of this complication in solid organ transplanted individuals or in people with diabetes receiving a GLP1RA based therapy. For this reason, and considering the powerful effect of GLP1RAs on cardiovascular protection beyond glycemic control, they should be proposed as first line therapy, as in type 2 diabetes. Particular caution should be taken in people with other risk factors for AP, such as a history of complex and repeated abdominal surgery.

## Data availability statement

The raw data supporting the conclusions of this article will be made available by the authors, without undue reservation.

## Ethics statement

The studies involving humans were approved by IRCCS Ca’ Granda – Ospedale Maggiore Policlinico Foundation. The studies were conducted in accordance with the local legislation and institutional requirements. The participants provided their written informed consent to participate in this study. Written informed consent was obtained from the individual(s) for the publication of any potentially identifiable images or data included in this article.

## Author contributions

VG: Conceptualization, Data curation, Investigation, Supervision, Writing – original draft, Writing – review & editing. IC: Data curation, Investigation, Methodology, Writing – original draft, Writing – review & editing. AG: Data curation, Writing – review & editing. VR: Data curation, Writing – review & editing. EO: Data curation, Methodology, Validation, Writing – review & editing.

## References

[1] Lankisch PG Apte M Banks PA. Acute pancreatitis. Lancet. (2015) 386:85–96. doi: 10.1016/S0140-6736(14)60649-8 25616312

[2] Working Group IAP/APA Acute Pancreatitis Guidelines. IAP/APA evidence-based guidelines for the management of acute pancreatitis. Pancreatology. (2013) 13:e1–15. doi: 10.1016/j.pan.2013.07.063 24054878

[3] Tenner S Baillie J DeWitt J Vege SS American College of Gastroenterology. American College of Gastroenterology guideline: management of acute pancreatitis. Am J Gastroenterol. (2013) 108:1400–15; 1416. doi: 10.1038/ajg.2013.218 23896955

[4] Arvanitakis M Dumonceau JM Albert J Badaoui A Bali MA Barthet M. Endoscopic management of acute necrotizing pancreatitis: European Society of Gastrointestinal Endoscopy (ESGE) evidence-based multidisciplinary guidelines. Endoscopy. (2018) 50:524–46. doi: 10.1055/a-0588-5365 29631305

[5] Forsmark CE Baillie J AGA Institute Clinical Practice and Economics Committee AGA Institute Governing Board. AGA Institute technical review on acute pancreatitis. Gastroenterology. (2007) 132:2022–44. doi: 10.1053/j.gastro.2007.03.065 17484894

[6] Rawla P Sunkara T Thandra KC Gaduputi V. Hypertriglyceridemia-induced pancreatitis: updated review of current treatment and preventive strategies. Clin J Gastroenterol. (2018) 11:441–8. doi: 10.1007/s12328-018-0881-1 29923163

[7] Kahaleh M Freeman M. Prevention and management of post-endoscopic retrograde cholangiopancreatography complications. Clin Endosc. (2012) 45:305–12. doi: 10.5946/ce.2012.45.3.305 PMC3429758 22977824

[8] Rosendahl J Witt H Szmola R Bhatia E Ozsvári B Landt O. (CTRC) variants that diminish activity or secretion are associated with chronic pancreatitis. Nat Genet. (2008) 40:78–82. doi: 10.1038/ng.2007.44 18059268 PMC2650829

[9] Rawla P Raj JP. Doxycycline-induced acute pancreatitis: A rare adverse event. Gastroenterol Res. (2017) 10:244–6. doi: 10.14740/gr838w PMC5593444 28912911

[10] Graham D Ito T Busuttil R Kaldas F. Pancreatitis in solid organ transplant patients: A review of the literature. OBM Hepatol Gastroenterol. (2019) 3. doi: 10.21926/obm.hg.1903029

[11] Wang XL Han W Zhao P Liu X Wang JZ Wang FR. Incidence, risk factors, outcomes, and risk score model of acute pancreatitis after allogeneic hematopoietic stem cell transplantation. Biol Blood Marrow Transplant. (2020) 26:1171–78. doi: 10.1016/j.bbmt.2019.12.721 31874219

[12] Bhadauria D Thammishetti V Goel A Kaul A Kushvah RS Behera M. An analysis of epidemiology, etiology, and outcomes of acute pancreatitis in renal transplant recipients. Transplant Proc. (2020) 52:865–72. doi: 10.1016/j.transproceed.2020.01.039 32146019

[13] Chuang YW Huang ST Yu TM Li CY Chung MC Lin CL. Acute pancreatitis risk after kidney transplantation: Propensity score matching analysis of a national cohort. PloS One. (2019) 14:e0222169. doi: 10.1371/journal.pone.0222169 31509567 PMC6738600

[14] Tabakovic M Salkic NN Bosnjic J Alibegovic E. Acute pancreatitis after kidney transplantation. Case Rep Transplant. (2012) 2012:768193. doi: 10.1155/2012/768193 23259142 PMC3504293

[15] Xu J Xu L Wei X Cao F Ou T Li F. A case report: acute pancreatitis associated with tacrolimus in kidney transplantation. BMC Nephrol. (2019) 20:209. doi: 10.1186/s12882-019-1395-x 31174507 PMC6555724

[16] Danalıoğlu A Mitchell OJ Singh VK Danalıoğlu AN Şentürk H Cameron AM. Acute pancreatitis following adult liver transplantation: A systematic review. Turk J Gastroenterol. (2015) 26:450–55. doi: 10.5152/tjg 26575039

[17] Camargo CA Jr. Greig PD Levy GA Clavien PA. Acute pancreatitis following liver transplantation. J Am Coll Surg. (1995) 181:249–56. 7545514

[18] Krokos NV Karavias D Tzakis A K Tepetes E Ramos S Todo. Acute pancreatitis after liver transplantation: incidence and contributing factors. Transpl Int. (1995) 8:1–7. doi: 10.1007/BF00366703 7534081 PMC2950630

[19] Russell TA Park S Agopian VG Zarrinpar A Farmer DG O'Neill S. Peritransplant pancreatitis: A marker of high mortality and graft failure in liver transplant patients. Liver Transpl. (2017) 23:925–32. doi: 10.1002/lt.24760 28294516

[20] Hujova A Macinga P Jarosova J Fronek J Taimr P Spicak J. Acute pancreatitis in patients after liver transplantation. Ann Transplant. (2022) 27:e938114. doi: 10.12659/AOT.938114 36523129 PMC9764668

[21] Marso SP Daniels GH Brown-Frandsen K Kristensen P Mann JF Nauck MA. Liraglutide and cardiovascular outcomes in type 2 diabetes. N Engl J Med. (2016) 375:311–22. doi: 10.1056/NEJMoa1603827 PMC4985288 27295427

[22] Pfeffer MA Claggett B Diaz R Dickstein K Gerstein HC Køber LV. Lixisenatide in patients with type 2 diabetes and acute coronary syndrome. N Engl J Med. (2015) 373:2247–57. doi: 10.1056/NEJMoa1509225 26630143

[23] Gerstein HC Colhoun HM Dagenais GR Diaz R Lakshmanan M Pais P. Dulaglutide and cardiovascular outcomes in type 2 diabetes (REWIND): a double-blind, randomised placebo-controlled trial. Lancet. (2019) 394:121–30. doi: 10.1016/S0140-6736(19)31149-3 31189511

[24] Marso SP Bain SC Consoli A Eliaschewitz FG Jódar E Leiter LA. Semaglutide and cardiovascular outcomes in patients with type 2 diabetes. N Engl J Med. (2016) 375:1834–44. doi: 10.1056/NEJMoa1607141 27633186

[25] Husain M Birkenfeld AL Donsmark M Dungan K Eliaschewitz FG Franco DR. Oral semaglutide and cardiovascular outcomes in patients with type 2 diabetes. N Engl J Med. (2019) 381:841–51. doi: 10.1056/NEJMoa1901118 31185157

[26] Hernandez AF Green JB Janmohamed S D'Agostino RB Sr Granger CB Jones NP. Albiglutide and cardiovascular outcomes in patients with type 2 diabetes and cardiovascular disease (Harmony Outcomes): a double-blind, randomised placebo-controlled trial. Lancet. (2018) 392:1519–29. doi: 10.1016/S0140-6736(18)32261-X 30291013

[27] American Diabetes Association Professional Practice Committee. 10. Cardiovascular disease and risk management: standards of care in diabetes-2024. Diabetes Care. (2024) 47:S179–218. doi: 10.2337/dc24-S010 PMC10725811 38078592

[28] US Food and Drug Administration. Exenatide (marketed as BYETTA): acute pancreatitis. Available online at: www.fda.gov/Drugs/DrugSafety/DrugSafetyNewsletter/ucm119034.htm#exenatide.

[29] Nauck MA. A critical analysis of the clinical use of incretin-based therapies: The benefits by far outweigh the potential risks. Diabetes Care. (2013) 36:2126–32. doi: 10.2337/dc12-2504 PMC3687264 23645884

[30] Butler PC Elashoff M Elashoff R Gale EA. A critical analysis of the clinical use of incretin-based therapies: Are the GLP-1 therapies safe? Diabetes Care. (2013) 36:2118–25. doi: 10.2337/dc12-2713 PMC3687282 23645885

[31] Gale E. Incretin therapy: should adverse consequences have been anticipated? BMJ. (2013) 346:f3617. doi: 10.1136/bmj.f3617 23751905

[32] Cohen D. Has pancreatic damage from glucagon suppressing diabetes drugs been underplayed? BMJ. (2013) 346:f3680. doi: 10.1136/bmj.f3680 23748128

[33] Kmietowicz Z. Potential harms of type 2 diabetes drugs have been ignored, finds BMJ investigation. BMJ. (2013) 346:f3782. doi: 10.1136/bmj.f3782 23757752

[34] Cohen D. Pressure mounts for companies to hand over data on antidiabetes drugs linked to pancreatic harm. BMJ. (2013) 346:f3900. doi: 10.1136/bmj.f3900 23775889

[35] Cohen D. European drugs agency clashes with scientists over safety of GLP-1 drugs. BMJ. (2013) 347:f4838. doi: 10.1136/bmj.f4838 23900829

[36] Li L Shen J Bala MM Busse JW Ebrahim S Vandvik PO. Incretin treatment and risk of pancreatitis in patients with type 2 diabetes mellitus: systematic review and meta-analysis of randomised and non-randomised studies. BMJ. (2014) 348:g2366. doi: 10.1136/bmj.g2366 24736555 PMC3987051

[37] Nreu B Dicembrini I Tinti F Mannucci E Monami M. Pancreatitis and pancreatic cancer in patients with type 2 diabetes treated with glucagon-like peptide-1 receptor agonists: an updated meta-analysis of randomized controlled trials. Minerva Endocrinol (Torino). (2023) 48:206–13. doi: 10.23736/S2724-6507.20.03219-8 32720500

[38] Levey AS Stevens LA Schmid CH Zhang YL Castro AF 3rd Feldman HI. A new equation to estimate glomerular filtration rate. Ann Intern Med. (2009) 150:604–12. doi: 10.7326/0003-4819-150-9-200905050-00006 PMC2763564 19414839

[39] Parenti DM Steinberg W Kang P. Infectious causes of acute pancreatitis. Pancreas. (1996) 13:356–71. doi: 10.1097/00006676-199611000-00005 8899796

[40] Lupo L Pirenne J Gunson B Nishimura Y Mirza DF Patapis P. Acute-pancreatitis after orthotopic liver transplantation. Transplant Proc. (1997) 29:473. doi: 10.1016/s0041-1345(96)00210-2 9123088

[41] Roberts MS Angus DC Bryce CL Valenta Z Weissfeld L. Survival after liver transplantation in the United States: a disease-specific analysis of the UNOS database. Liver Transpl. (2004) 10:886–97. doi: 10.1002/lt.20137 15237373

[42] Verran DJ Gurkan A Chui AK Dilworth P Koorey D McCaughan G. Pancreatitis in adult orthotopic liver allograft recipients: risk factors and outcome. Liver Transpl. (2000) 6:362–6. doi: 10.1053/lv.2000.5203 10827240

[43] Yang AL McNabb-Baltar J. Hypertriglyceridemia and acute pancreatitis. Pancreatology. (2020) 20:795–800. doi: 10.1016/j.pan.2020.06.005 32571534

